# Rifaximin use favoured micafungin-resistant *Candida* spp. infections in recipients of allogeneic hematopoietic cell transplantation

**DOI:** 10.1007/s00277-021-04569-x

**Published:** 2021-06-28

**Authors:** Francesca Marzuttini, Antonella Mancusi, Samanta Bonato, Mario Griselli, Sara Tricarico, Genni Casarola, Matteo Paradiso, Loredana Ruggeri, Adelmo Terenzi, Mara Merluzzi, Anna Prigitano, Anna Maria Tortorano, Lucia Pitzurra, Brunangelo Falini, Alessandra Carotti, Andrea Velardi, Antonio Pierini

**Affiliations:** 1 Division of Hematology and Clinical Immunology, University of Perugia, Perugia, Italy; 2 Department of Biomedical Sciences for Health, Università Degli Studi Di Milano, Milan, Italy; 3 Division of Microbiology, University of Perugia, Perugia, Italy

**Keywords:** Candida, Rifaximin, Hematopoietic stem cell transplantation, Microbiome, Prophylaxis, Echinocandin

## Abstract

Damage to gut mucosa following conditioning regimens may favour bacterial infections that can trigger graft versus host disease (GvHD) in patients undergoing allogeneic hematopoietic stem cell transplantation (HSCT). Rifaximin, an oral and non-absorbable antibiotic, has been recently proposed as effective prophylaxis to reduce bacterial infections in the gut and consequently acute GvHD in this setting. The present study evaluated safety and outcomes of HSCT patients that were treated with rifaximin prophylaxis at Perugia University Hospital. Rifaximin prophylaxis was introduced as standard of care in HSCT patients in May 2018. We retrieved data from 118 consecutive transplants, and we compared the outcomes of rifaximin-treated patients with historical controls that did not receive antibiotic prophylaxis. While incidences of neutropenic fever, documented bacterial infections, and aGvHD were similar in the two groups, we found an increased frequency of invasive candidiasis and clinically relevant *Candida* spp. infections in rifaximin-treated patients (5 patients vs 1 patient, 25% [± 0.99%] vs 1% [± 0.01%], p < .0001). Three rifaximin-treated patients experienced life-threating candidemia (2 *C. krusei*, 1 *C. orthopsilosis*). Rifaximin was the only factor that increased the risk of *Candida* spp. infections. Rifaximin could have contributed to microbiome disruption which favoured an outbreak of life-threatening *Candida* infections. This important complication forced us to halt its use. Larger, prospective studies are needed to assess the impact of rifaximin prophylaxis on incidence of bacterial infections, aGvHD, and survival of HSCT patients.

**Supplementary Information:**

The online version contains supplementary material available at 10.1007/s00277-021-04569-x.

## Introduction

Bacterial infections are common complications during neutropenia that follows allogeneic hematopoietic stem cell transplantation (HSCT), but there is no general consensus regarding the need, the type, and the duration of antibiotic prophylaxis in this setting. Antibiotic prophylaxis with fluoroquinolones alone or fluoroquinolones plus metronidazole has been considered a standard of care at most institutions as it has been shown to reduce incidence of gram-negative sepses [1, 2]. However, recent studies showed an increase of bacterial resistance to fluoroquinolones which questioned their efficacy [3]. Thus, some centres even abandoned prophylaxis use and opted for a pre-emptive or empiric approach to bacterial infections.

Recent studies show that intestinal microbiota regulates immune homeostasis. Loss of microbiota diversity and prevalence of enterococci have been linked to the onset of acute graft-versus-host disease (aGvHD), an immune-mediated and potentially life-threatening complication of HSCT [4, 5]. Rifaximin, an oral and almost non absorbable antibiotic which preserves intestinal microbiota diversity, can reduce enterococci and may exert anti-inflammatory activities. A single-centre retrospective study by Weber et al. suggested that antibiotic prophylaxis with rifaximin could reduce incidence of aGvHD and improve overall survival of transplanted patients if compared to fluoroquinolone plus metronidazole prophylaxis [6].

 Our group historically chose to avoid antibiotic prophylaxis for HSCT patients because of high prevalence of fluoroquinolone-resistant bacterial infections. Following the study by Weber et al., we recently changed this policy and introduced antibiotic prophylaxis with rifaximin to reduce such infections and, possibly, incidence of aGvHD. Here we report incidence of infections and outcomes of patients that received rifaximin prophylaxis at our institution.

## Materials and methods

### Ethics

The study was conducted according to the revised Helsinki Declaration and was approved by Umbria Regional Hospital Institutional Review Board (IRB). Written informed consent was obtained from each patient.

### Demographics

In this retrospective study, we compared outcomes of rifaximin-treated patients with historical controls at our institution. We analysed transplants performed from January 2016 to August 2019 at the University Hospital of Perugia.

We started rifaximin use in May 2018. Rifaximin 200 mg q12h was given from the beginning of the conditioning regimen to day + 20 post-transplant. Controls received no antibiotic prophylaxis. All patients without pre-HSCT fungal disease received micafungin 50 mg q24h as antifungal primary prophylaxis, while patients who experienced possible or probable fungal pneumonia before transplant received lyposomal amphotericin-B (L-AmB) 1–3 mg/kg q24h as secondary prophylaxis. Trimethoprim-sulfamethoxazole 960 mg three times each week was used as prophylaxis for *P. jirovecii* pneumonia starting at day +50 after transplant.

We retrieved the following information for each patient: age, gender, underlying haematologic disease, type of graft (HLA-matched related HSCT, HLA-mismatched related, unrelated), intensity of the conditioning regimen, previous *Candida* spp. colonization, diagnosis of fungal pneumonia before HSCT, previous exposure to echinocandins, antifungal prophylaxis, and duration of micafungin prophylaxis (Table 1). We used the EORTC (European Organization for Research and Treatment of Cancer) criteria to define invasive candidiasis [7]. *Candida* spp. isolates that were considered worth of systemic antifungal treatment because they were associated to specific clinical signs and/or symptoms of disease have been defined as clinically relevant *Candida* spp. infections and were included in the analysis.

**Table 1:** Demographics of the patient population. *HLA*, human leukocyte antigen; *HSCT*, allogeneic hematopoietic stem cell transplantation; *L-AmB*, lyposomal amphotericin-B

	Rifaximin	No prophylaxis	*p* value	All patients
No of patients	21 (18%)	97 (82%)		118
Age (median)	57	52	.031	52.5
Gender (male)	7 (33%)	65 (67%)	.004	72 (61%)
Underlying hematologic disease			.68	
Myeloid	12 (57%)	60 (62%)		72 (61%)
Lymphoid	9 (43%)	37 (38%)		46 (39%)
Type of graft			.42	
HLA-matched related	3 (14%)	20 (21%)		23 (20%)
HLA-mismatched related	18 (86%)	72 (74%)		90 (76%)
Unrelated	0	5 (5%)		5 (4%)
Conditioning regimen			.77	
Myeloablative	17 (81%)	81 (84%)		98 (83%)
Reduced-intensity or non-myeloablative	4 (19%)	16 (16%)		20 (17%)
Previous *Candida *spp. colonization	8 (38%)	40 (41%)	.79	48 (41%)
Fungal pneumonia before HSCT	3 (14%)	25 (26%)	.26	28 (24%)
Previous exposure to echinocandins	3 (14%)	9 (9%)	.49	12 (10%)
Antifungal prophylaxis			.009	
Primary (with micafungin)	21 (100%)	72 (74%)		93 (79%)
Secondary (with L-AmB)	0	25 (26%)		25 (21%)
Duration of micafungin prophylaxis (median days, range)	28 (18–102)	28 (3–72)	.37	28 (3–102)

### Statistical analysis

Demographics and prognostic variables were compared using the χ^2^ test for categorical variables, and the Student t test or Mann–Whitney test for continuous variables. The Kaplan–Meier method evaluated overall and disease-free survival. A log-rank test assessed rifaximin impact on survival. Cumulative incidence (CI) estimates were used for relapse and non-relapse mortality (NRM) because they were considered competing risks. CI of aGvHD or *Candida* infections was calculated using death from any cause as competing risk. Gray test compared impact of rifaximin on univariate competing risk outcomes. Multivariate analyses assessed the impact of rifaximin on incidence of clinically relevant *Candida* spp. infections. Different demographic features (Table 1) were included as variables using a Cox regression model in a conditional forward stepwise fashion to identify factors with a significant impact on outcomes. All p values were 2-sided and considered significant at p < 0.05.

## Results

We retrieved data from 118 consecutive transplants. Twenty-one received rifaximin, while 97 received no antibiotic prophylaxis. Engraftment occurred in all, but in six patients (1 received rifaximin, 5 no antibiotic prophylaxis, p = not significant, NS). Patients were well-matched for underlying hematologic disease, type of graft, intensity of the conditioning regimen, previous *Candida* spp. colonization, diagnosis of fungal pneumonia before HSCT, previous exposure to echinocandins, and duration of micafungin prophylaxis. Patients who received rifaximin were slightly older than patients in the control group, and female/male ratio was slightly higher. None of the 21 patients who received rifaximin was treated with L-AmB as antifungal prophylaxis, while L-AmB was used as secondary antifungal prophylaxis in 25 control transplants. Patient characteristics are detailed in Table 1.

**Fig. 1:**
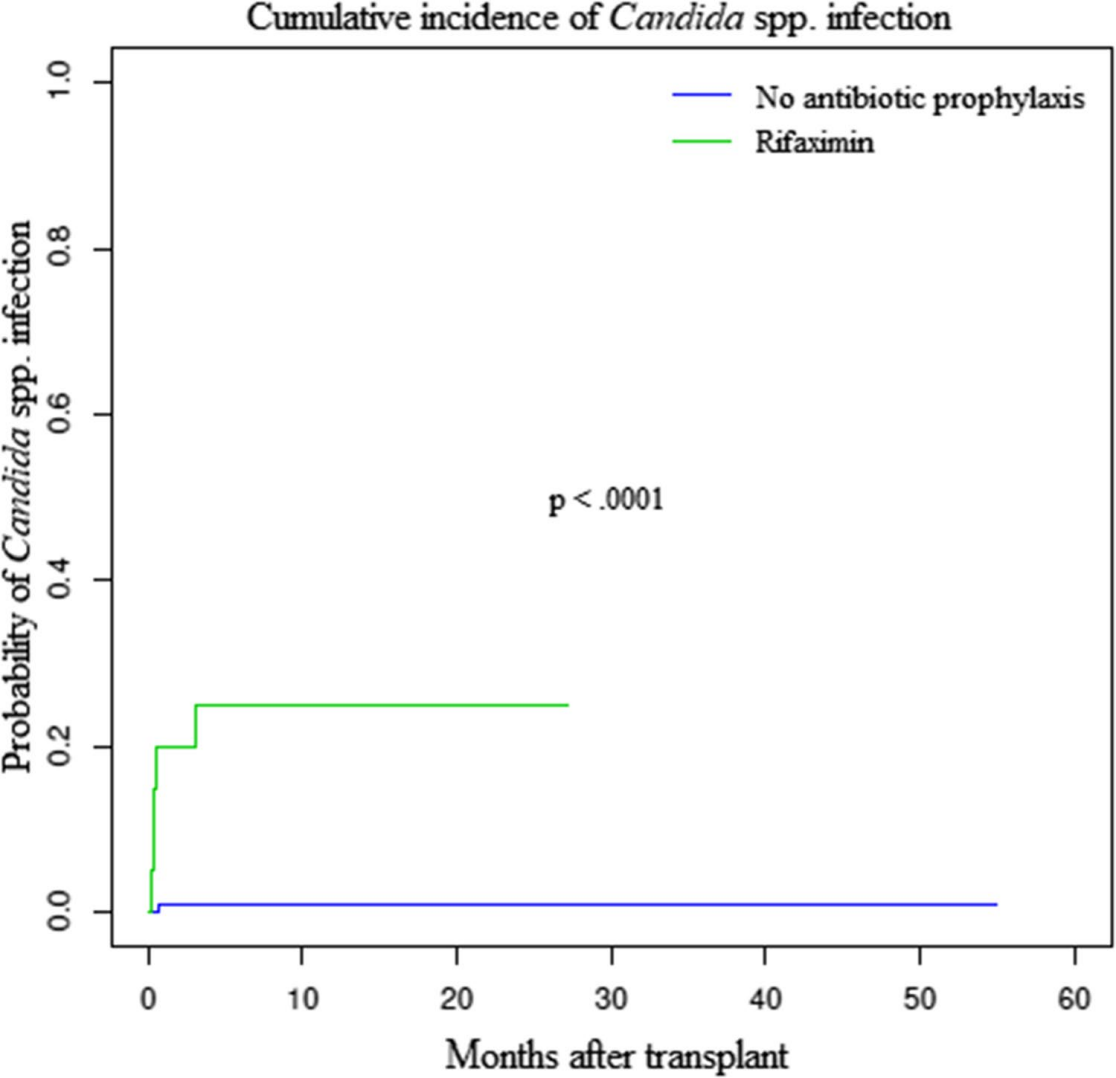
Cumulative incidence of clinically relevant *Candida* spp. infections. Incidence of clinically relevant *Candida* spp. infections was higher in rifaximin-treated patients (25% [± 0.99%] vs 1% [± 0.01%])

Incidences of neutropenic fever (100% in rifaximin-treated patients vs 96% in control patients, p = NS), documented sepsis (25% vs 33%, p = NS), and use of carbapenems (80% vs 75%, p = NS) were similar between rifaximin-treated patients and historical controls (Table 2). Also, aGvHD occurred with a similar incidence between the two groups (CI in rifaximin-treated patients: 35% [± 1.22%]; CI in control patients: 35% [± 0.25%], p = NS) (supplementary Fig. 1). Surprisingly, we found clinically relevant *Candida* spp. infections (5 patients vs 1, 25% [± 0.99%] vs 1% [± 0.01%], p < 0.0001) (Fig. 1) and invasive candidiases (3 vs 0, 15% [± 0.67%] vs 0% [± 0%], p = 0.0002) (supplementary Fig. 2) were more frequent in rifaximin-treated patients. Incidences of other fungal infections and viral (Cytomegalovirus, CMV and Epstein-Barr virus, EBV) reactivations did not differ in rifaximin-treated patients and controls (other fungal infections: 30% vs 43%, p = NS; CMV: 55% vs 59%, p = NS; EBV: 25% vs 36%, p = NS). Three of the 5 rifaximin-treated patients with *Candida* spp. infection experienced life-threatening invasive candidiasis (3 candidaemias, 2 of which by *C. krusei*, 1 by *C. orthopsilosis*). Details about *Candida* spp. isolates are reported in supplementary table 1. All *Candida* infections happened early after transplant (medium time 26 days, range 5–91). Multivariate analysis confirmed rifaximin was the only factor that increased the risk of *Candida* spp. infection as such risk was not dependent upon age, gender, underlying hematologic disease, type of graft, intensity of the conditioning regimen, and previous *Candida* spp. colonization (supplementary table 2). To assess whether antifungal prophylaxis had an impact on *Candida* spp. infections, patients that received secondary prophylaxis with L-Amb were excluded from the analysis as none of them was treated with rifaximin. Rifaximin prophylaxis was confirmed as the only risk factor for the development of invasive candidiasis and clinically relevant *Candida* spp. infection (supplementary Fig. 3).

**Table 2:** Outcomes of transplanted patients according to the use of rifaximin prophylaxis. Percentage of events is calculated on the total of engrafted patients. *HSCT*, allogeneic hematopoietic stem cell transplantation; *CMV*, Cytomegalovirus; *EBV*, Epstein-Barr virus; *GvHD*, graft versus host disease

	Rifaximin	No prophylaxis	*p* value	All patients
Engraftment	20/21 (95%)	92/97 (95%)	.94	112/118 (95%)
Neutropenic fever	20 (100%)	88 (96%)	.34	108 (96%)
Documented sepses	5 (25%)	30 (33%)	.51	35 (31%)
Bacterial isolate				
*Escherichia coli*	2 (40%)	16 (53%)	.41	18 (51%)
*Enterococcus fecium* and *faecalis*	0	2 (6%)	.51	2 (6%)
Other gram positive	2 (40%)	10 (33%)	.91	12 (34%)
Other gram negative	1 (20%)	2 (6%)	.48	3 (9%)
Use of carbapenems	16 (80%)	69 (75%)	.64	85 (76%)
Fungal infections post HSCT				
Clinically relevant candidiasis	5 (25%)	1 (1%)	** < *.0001***	6 (5%)
Invasive candidiasis	3 (15%)	0 (0%)	***.0002***	3 (3%)
Other	6 (30%)	40 (43%)	.27	46 (41%)
Viral reactivations				
CMV	11 (55%)	54 (59%)	.76	65 (58%)
EBV	5 (25%)	33 (36%)	.35	38 (34%)
Acute GvHD				
Grade II–IV	7 (35%)	32 (35%)	.98	39 (35%)
Grade III–IV	4 (20%)	21 (23%)	.78	25 (22%)
Steroid refractory	3 (15%)	19 (21%)	.42	22 (20%)

L-AmB was chosen for the treatment of micafungin-resistant *Candida* infections in 5 of the 6 patients, while voriconazole was used to treat one of them. All the patients resolved the *Candida* spp. infections. *Candida* infections did not impact on disease-free survival (55% in rifaximin-treated patients vs 57% in patients that received no antibiotic prophylaxis, p = NS) (supplementary Fig. 4) and overall survival (60% vs 61%, p = NS) (supplementary Fig. 5) probably because of the low number of rifaximin-treated patients. In fact, the increase of clinically relevant *Candida* infections forced us to halt rifaximin use.

## Discussion

The present study shows rifaximin prophylaxis could favour *Candida* spp. infections in patients who undergo allogeneic HSCT and are treated with echinocandin-based antifungal prophylaxis. The low number of patients that were treated with rifaximin prophylaxis at our institution does not allow us to evaluate its efficacy in preventing bacterial infections and, consequently, its possible impact on incidence of aGvHD. Nevertheless, the rise of *Candida* spp. infections in this small subset of patients and the clinical impact of such complications urged us to quickly modify our anti-infective prophylactic strategies in allogeneic HSCT recipients. The present report underlines the clinical need to adjust anti-infective prophylaxes to local epidemiology taking into account drug interactions and concomitant effects on microbiome. Moreover, the present study demonstrates the clinical relevance of fungal dysbiosis in the context of HSCT and urges the scientific community to explore the impact of fungal microbiota on HSCT outcomes. In this retrospective study, we could not investigate whether rifaximin impacted on microbiome diversity. However, despite that the rate of bacterial (e.g. *E. coli* and enterococci) infections did not differ between rifaximin-treated patients and controls, it is likely that rifaximin played a relevant role in modifying environmental factors that favoured this *Candida* outbreak in echinocandin-treated patients. Indeed, we did not observe further *Candida* spp. infections after rifaximin use was suspended in patients that received the same micafungin prophylaxis regimen.

In fact, all the 6 *Candida* spp. infections occurred in patients that were receiving prophylactic micafungin. Development of echinocandin resistance in *Candida* spp. infection is a rare phenomenon. It is probably favoured by prolonged echinocandin exposure and biofilm formation which determines poor drug penetration and consequently strong selective pressure [8, 9]. The mechanism of acquired resistance to echinocandin therapy involves genetic mutations in specific hot spot regions of *fks* target genes, and it is usually associated with an increase in echinocandin minimum inhibitory concentration (MIC) in vitro [8]. Echinocandin MIC elevation, response to echinocandin in vivo, and clinical outcomes may be different according to the type of *fks1* mutation [10]. Thus, we analysed the genotype of the two *C. krusei* isolates and we found that both presented the L701M mutation in the *fks1* gene. While other *fks1* mutations are clearly associated to echinocandin resistance, the role of L701M mutation is still unclear. This mutation has been previously identified in association with elevated caspofungin MIC if coupled to other mutations with an ascertained role in echinocandin resistance [11, 12]. On the other hand, L701M mutation alone may not have an impact on MIC values [13]. In fact, all our isolates were sensitive to echinocandins at the antifungal susceptibility testing while they showed resistance to prophylactic doses of micafungin in vivo. Thus, it is possible that L701M mutation may favour in vivo resistance to relatively low dose echinocandin regimens, at least in *Candida krusei* infections. Weber et al. did not specify the antifungal prophylaxis used and did not report the rate of Candida infections in their study [6]. Indeed, safety of rifaximin in HSCT recipients needs to be explored when coupled with antifungal prophylaxis other than micafungin.

In conclusion, despite that our study retrieved data from a relatively low number of patients with a possible bias due to local epidemiology and to a specific strategy in antimicrobial prophylaxis and treatment, it shows prophylactic use of rifaximin in HSCT patients may result in an increase of *Candida* spp. infections. Further and wider studies are required to investigate the role of rifaximin in the modulation of microbiota diversity, the development of bacterial and *Candida* infections, and its final effect on patients undergoing HSCT.

## Supplementary Information

Below is the link to the electronic supplementary material.

Supplementary file1 (PDF 935 KB)

## Data Availability

The authors will provide data after verified and detailed institutional request by professional members. Data will be available starting 3 months within 1 year from the date of publication. Request needs to be made to antonio.pierini@unipg.it.
