# Supplementary material for: Rifaximin use favoured micafungin-resistant Candida spp. infections in recipients of allogeneic hematopoietic cell transplantation
Source: Ann Hematol. 2021 Jun 28;100(9):2375–80. doi: 10.1007/s00277-021-04569-x (PMC8357665; doi:10.1007/s00277-021-04569-x)
Supplement: Supplementary file 1 — Supplementary file1 (PDF 935 KB) [file 277_2021_4569_MOESM1_ESM.pdf]

Rifaximin use favoured micafungin resistant *Candida* spp. infections in recipients of allogeneic hematopoietic cell transplantation.

Running title: *Candida* outbreak following rifaximin use.

Francesca Marzuttini[1], Antonella Mancusi[1], Samanta Bonato[1], Mario Griselli[1], Sara Tricarico[1], Genni Casarola[1], Matteo Paradiso[1], Loredana Ruggeri[1], Adelmo Terenzi[1], Mara Merluzzi[1], Anna Prigitano[2], Anna Maria Tortorano[2], Lucia Pitzurra[3], Brunangelo Falini[1], Alessandra Carotti[1], Andrea Velardi[1], Antonio Pierini[1]

[1]Division of Hematology and Clinical Immunology, University of Perugia, Perugia, Italy, [2]Department of Biomedical Sciences for Health, Università degli Studi di Milano, Milan, Italy, [3]Division of Microbiology, University of Perugia, Perugia, Italy

Corresponding author:
Antonio Pierini, MD, PhD
Centro di Ricerca Emato-Oncologico CREO,
University of Perugia, Piazzale Menghini 1, 06129, Perugia, Italy
e-mail: antonio.pierini@unipg.it
tel: +393355477611

	Patient 1	Patient 2	Patient 3	Patient 4	Patient 5	Patient 6
**Rifaximin prophylaxis**	No	Yes	Yes	Yes	Yes	Yes
**Isolate**	*Candida lusitaniae*	*Candida krusei*	*Candida krusei*	*Candida orthopsilosis*	*Candida orthopsilosis*	*Candida parapsilosis*
**Clinical picture**	Pneumonia	Candidemia	Candidemia	Candidemia	Pneumonia	Urinary infection
**Days after transplant**	21	10	12	5	17	91
**Antifungal susceptibility testing- MIC**	Micafungin: <=0,06<br>Caspofungin: <=0,25<br>L-AmB: <=0,25<br>Voriconazole: <=0,12<br>Fluconazole: <=1<br>Isavuconazole: not evaluated	Micafungin: 0,060<br>Caspofungin: 0,060<br>L-AmB: 0,500<br>Voriconazole: 0,120<br>Fluconazole: 128<br>Isavuconazole: 0,032	Micafungin: 0,060<br>Caspofungin: 0,060<br>L-AmB: 0,250<br>Voriconazole: 0,250<br>Fluconazole: 32<br>Isavuconazole: 0,047	Micafungin: 0,250<br>Caspofungin: 0,250<br>L-AmB: 0,125<br>Voriconazole: 0,008<br>Fluconazole: 0,500<br>Isavuconazole: 0,032	Micafungin: 0,250<br>Caspofungin: 0,500<br>L-AmB: 1,000<br>Voriconazole: 0,030<br>Fluconazole: 1,000<br>Isavuconazole: not evaluated	Micafungin: 1,000<br>Caspofungin: 0,500<br>L-AmB: 0,500<br>Voriconazole: 0,015<br>Fluconazole: 2,000<br>Isavuconazole: not evaluated
**Treatment**	L-Amb	L-Amb	L-Amb	L-Amb	L-Amb	Voriconazole
**Outcome**	Resolved	Resolved	Resolved	Resolved	Resolved	Resolved

Supplementary table 1. Characteristics of clinically relevant *Candida* infections. MIC = minimum inhibitory concentration; L-AmB = lyposomal amphotericin-B.

	Clinically relevant *Candida* spp. infections		
**Variables**	**HR**	**CI**	**p value**
**Age**	1,013	,938-1,093	,747
**Gender (male)**	,598	,083-4,283	,609
**Underlying hematologic disease**	,140	,003-5,857	,302
**Type of graft**	1,076	,072-15,968	,958
**Conditioning regimen**	,069	,002-2,518	,145
**Previous *Candida spp* colonization**	,681	,110-4,206	,679
**Rifaximin**	36,950	3,579-381,460	,002

Supplementary table 2: Multivariate analysis for clinically relevant *Candida* spp. infections. HR=hazard ratio; CI= confidence interval.

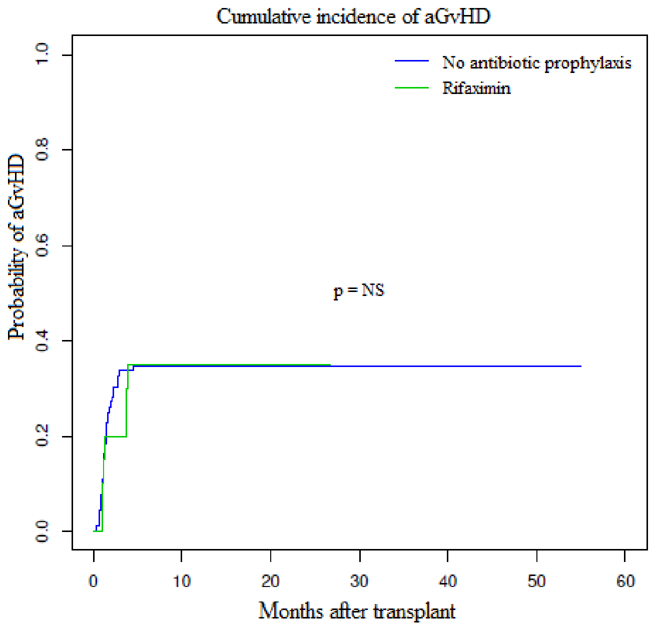

Supplementary figure 1. Cumulative incidence of aGvHD. aGvHD was similar between rifaximin-treated patients and controls (35% [±1.22%] vs 35% [±0.25%]). NS = not significant.

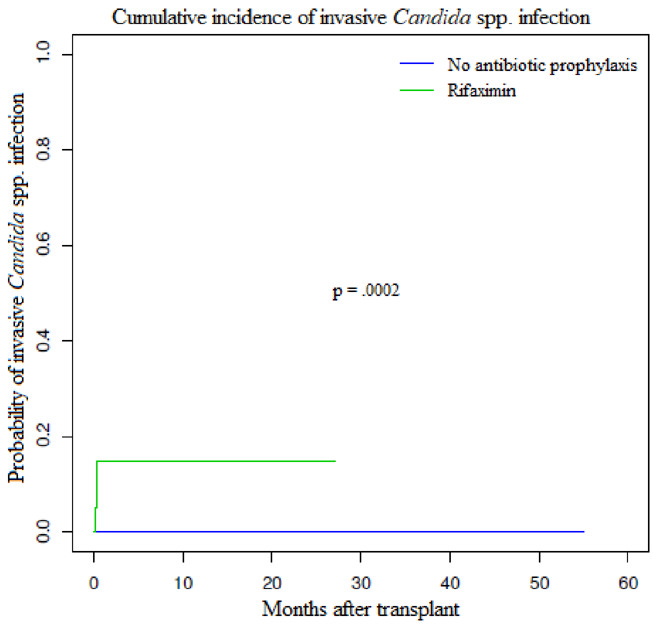

Supplementary figure 2. Cumulative incidence of invasive *Candida* spp. infections. Incidence of invasive *Candida* spp. infections was higher in rifaximin treated patients (15% [±0.67%] vs 0% [±0%]).

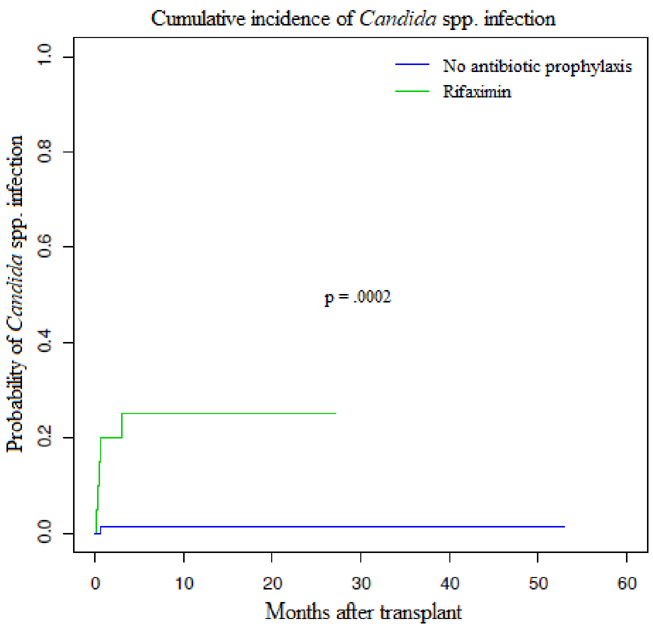

Supplementary figure 3. Cumulative incidence of clinically relevant *Candida* spp. infections in patients that received primary prophylaxis with micafungin. In this subanalysis patients that received secondary prophylaxis with L-Amb were excluded as none of them was treated with rifaximin. Rifaximin prophylaxis was confirmed as the only risk factor for the development of clinically relevant *Candida* spp. infections (25% [±0.99%] vs 1,3% [±0.02%]).

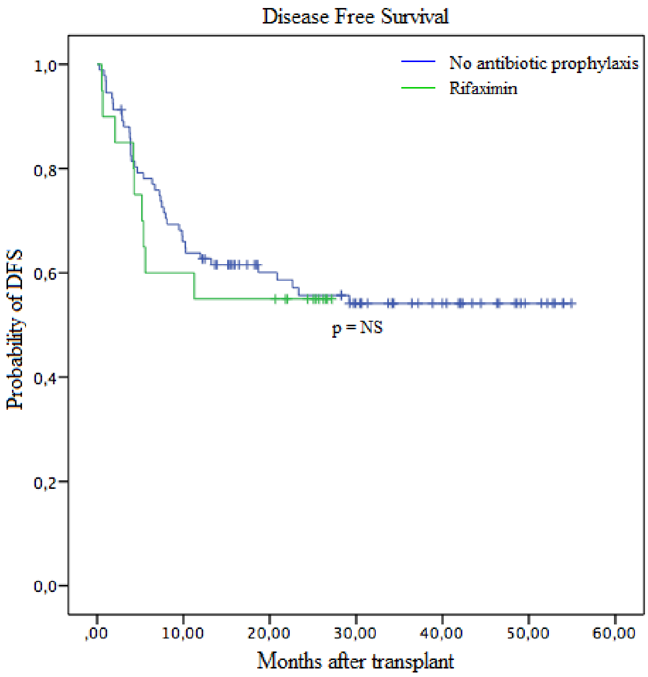

Supplementary figure 4. Disease free survival. Disease free survival was similar between rifaximin-treated patients and controls (55% rifaximin vs 57% no antibiotic prophylaxis, p=NS). DFS = disease free survival.

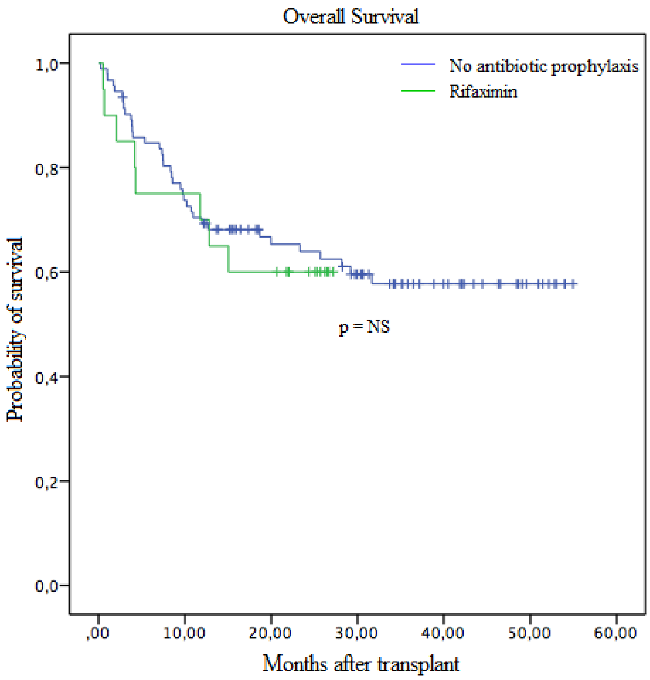

Supplementary figure 5. Overall survival. Overall survival was similar between rifaximin-treated patients and controls (60% rifaximin vs 61% no antibiotic prophylaxis, p=NS).
